# Using A-Mode Ultrasound to Assess the Body Composition of Soccer Players: A Comparative Study of Prediction Formulas

**DOI:** 10.3390/diagnostics13040690

**Published:** 2023-02-12

**Authors:** Paul Muntean, Monica Neagu, Elena Amaricai, Horia G. Haragus, Roxana Ramona Onofrei, Adrian Neagu

**Affiliations:** 1 Department of Functional Sciences, Victor Babes University of Medicine and Pharmacy Timisoara, 300041 Timisoara, Romania; 2 Center for Modeling Biological Systems and Data Analysis, Victor Babes University of Medicine and Pharmacy Timisoara, 300041 Timisoara, Romania; 3 Department of Rehabilitation, Physical Medicine and Rheumatology, Research Center for Assessment of Human Motion, Functionality and Disability, Victor Babes University of Medicine and Pharmacy Timisoara, 300041 Timisoara, Romania; 4 Department of Orthopedics, Victor Babes University of Medicine and Pharmacy Timisoara, 300041 Timisoara, Romania; 5 Department of Physics and Astronomy, University of Missouri, Columbia, MO 65211, USA

**Keywords:** body fat percentage, methods comparison, Bland–Altman analysis, Passing–Bablok regression

## Abstract

For elite athletes, monitoring body composition is important for maximizing performance without health risks. Amplitude (A)-mode ultrasound (AUS) has attracted increasing attention as an alternative to skinfold thickness measurements commonly used for assessing the amount of body fat in athletes. AUS accuracy and precision, however, depend on the formula used to predict body fat percentage (%BF) from subcutaneous fat layer thicknesses. Therefore, this study evaluates the accuracy of the 1-point biceps (B1), 9-sites Parrillo, 3-sites Jackson and Pollock (JP3), and 7-sites Jackson and Pollock (JP7) formulas. Relying on the previous validation of the JP3 formula in college-aged male athletes, we took AUS measurements in 54 professional soccer players (aged 22.9 ± 3.83 y, mean ± SD) and compared the results given by different formulas. The Kruskal–Wallis test indicated significant differences (*p* < 10^−6^), and Conover’s post hoc test revealed that the JP3 and JP7 data come from the same distribution, whereas the data given by B1 and P9 differ from all the others. Lin’s concordance correlation coefficients for B1 vs. JP7, P9 vs. JP7, and JP3 vs. JP7 were 0.464, 0.341, and 0.909, respectively. The Bland–Altman analysis indicated mean differences of −0.5 %BF between JP3 and JP7, 4.7 %BF between P9 and JP7, and 3.1 %BF between B1 and JP7. This study suggests that JP7 and JP3 are equally valid, whereas P9 and B1 overestimate %BF in athletes.

## 1. Introduction

Body composition tracking is increasingly popular with people interested in a healthy lifestyle. While it is useful for the general population, it is highly recommended for professional athletes [1] and patients who need body mass management to cope with the adverse effects of obesity, metabolic disorders, and aging [2].

Athletes often follow unhealthy weight loss regimens in an attempt to boost their performance by losing body fat. Diets that aim at rapid weight loss, however, can result in severe dehydration and eating disorders. Even in the absence of these complications, extreme dieting can be harmful. Although it is a mechanically passive weight to carry, adipose tissue is essential to general health due to its endocrine functions. Therefore, the periodic evaluation of an athlete’s body composition is important for implementing a healthy and effective training program [3]. In soccer, for example, body composition and fitness measures are currently used to characterize the evolution of young soccer players. Body composition and physical fitness were found to depend on age group and playing positions and improved during the maturation of the player [4].

Body composition assessment based on skinfold thickness measurements has long been the method of choice because it is convenient and robust against prior exercise, food intake, and changes in hydration level. Remarkably, these advantages have been reconfirmed by a recent study, which compared the skinfold thickness method with bioelectrical impedance analysis (BIA), ultrasound, and highly regarded laboratory techniques, including underwater weighing, air displacement plethysmography (ADP), three-dimensional (3D) optical scanning, and dual-energy x-ray absorptiometry (DXA) [5]. Nevertheless, the Achilles’ heel of the skinfold thickness method is technician training. It takes time and carefully supervised exercise to train anthropometry technicians capable of high inter-rater reliability in skinfold thickness measurements [6,7].

A-mode ultrasound (AUS) is a portable and relatively affordable alternative to the skinfold thickness method [8]. It is equally insensitive to hydration status [9] and requires less technician training than skinfold thickness measurement. Indeed, when undergraduate Kinesiology students with minimal (25 min.) training in both AUS and skinfolds performed repeated tests, their inter-rater reliability in %BF assessments of male subjects was significantly higher for AUS than for skinfolds—as quantified by intraclass correlation coefficients (ICCs) equal to 0.990 and 0.862, respectively [10]. By contrast, experienced technicians obtained similar ICCs in %BF assessments by both techniques (0.987 for AUS and 0.966 for skinfolds) [11]. The relatively high inter-rater reliability demonstrated by novice examiners was attributed, in part, to a double-check of measurement site locations by experienced technicians. Thus, a major source of variability was eliminated from both the AUS and skinfold-based tests [10]. The highest test–retest reliability recorded to date with the AUS technique (ICC = 0.996) was achieved when expert technicians located the measurement sites and marked them using surgical markers and, occasionally, assisted the instrument’s algorithm in identifying the fat–muscle interface [11]. Nevertheless, even in full-automatic mode and in the absence of marked sites, the test–retest reliability of AUS was excellent (ICC = 0.979) [12].

AUS was validated in male athletes against a well-established reference technique, ADP. Using the BodyMetrix System (IntelaMetrix, Brentwood, CA, USA) with the 3-sites Jackson and Pollock (JP3) formula implemented in the software shipped with the ultrasound device, Wagner et al. found that AUS was within ±1.5 %BF of ADP for male athletes but overestimated the adiposity of female athletes by 3.0–5.5 %BF [11].

Percent body fat estimates by the BodyMetrix instrument with the JP3 formula, BIA, and ADP were deemed valid in a study conducted on 26 college-aged subjects (18 men and 8 women) [13]. The BodyMetrix System with the 7-sites Jackson and Pollock (JP7) formula was found to underestimate %BF in obese and overweight subjects (20 men and 27 women) [14], whereas, in a mainly normal-weight sample of the general population (16 men and 33 women), the mean difference between AUS (using the JP7 formula) and ADP was insignificant,−0.32 %BF [15].

Our reliability study [12] indicated that, at least in automatic mode, the JP7 formula assures higher reliability than JP3—the formula validated in college-aged male athletes [11]. JP3 and JP7 were found in good agreement in a vast investigation of all the formulas included in the BodyMetrix instrument’s software for assessing the global adiposity of men [16]. Lowry et al. used the ADP and 9 AUS formulas to measure %BF in a sample of 42 men of diverse body types, between 18 and 65 years of age. Compared to ADP, both JP3 and JP7 resulted in a slight, statistically insignificant underestimation of %BF. The latter, however, had the lowest standard error of estimate and total error [16]. Therefore, we asked the question of whether the JP7 formula applied to an athletic population would also give a valid assessment of %BF.

Besides JP3 and JP7, professional athletes also value the 9-sites Parrillo (P9) formula, initially developed for tracking the body composition of bodybuilders via skinfold thickness measurements [17]. The P9 formula is based on a thorough sampling of subcutaneous adipose tissue thicknesses, including six of the sites considered by JP7. In their heterogeneous group of men, however, Lowry et al. found a statistically significant mean difference of −4.72 %BF between ADP and P9 (i.e., P9 overestimated adiposity). Furthermore, the 1-point biceps formula (B1) is also appealing because it is convenient both for the subject and the examiner [18].

Thus, the goal of the present study was to compare the predictions of the JP7, JP3, P9, and B1 formulas in a sample of male soccer players. Analyzed with several statistical methods, the results confirm the hypothesis that JP3 and JP7 give equally valid assessments of %BF, which differ significantly from the ones given by P9 and B1.

## 2. Materials and Methods

This study was performed in agreement with the ethical principles stipulated in the Declaration of Helsinki [19]. The athletes who volunteered for the study provided written informed consent, and the study protocol was approved by the Ethics Committee of our institution (approval no. 20/24 July 2019). The participants satisfied all of the following inclusion criteria: (i) elite soccer players affiliated with a professional soccer club, (ii) participation in soccer competitions, and (iii) soccer training at least four times a week.

The volunteers were not considered for this investigation if any of the following exclusion criteria were met: (i) age under 18 years, (ii) medical records with chronic diseases, (iii) diagnosed with acute infections, or (iv) injuries suffered during the last 3 months.

### 2.1. Study Population

Fifty-four male soccer players were included in this study. Table 1 lists their demographic data.

### 2.2. A-Mode Ultrasound Measurements

In this study, we used a BodyMetrix BX2000 ultrasound device (IntelaMetrix, Brentwood, CA, USA), henceforth referred to as BodyMetrix, operating in A-mode at a frequency of 2.5 MHz. All the measurements were taken by the same examiner (a Sports Medicine resident with 2 years of experience in AUS).

Body mass was measured to the nearest 0.1 kg using a Tanita HD-384 digital scale (Tanita Europe, Amsterdam, The Netherlands). A wall-mounted tape measure (GIMA 27335, GIMA, Gessate, Italy) was used to measure stature to the nearest 0.5 cm.

For each participant, we built a new client profile in the BodyViewProFit software (version 2.1.0.9045), which included their name, age, gender, height, weight, and athletic type. To assist BodyViewProFit in detecting the interface between subcutaneous fat and the underlying muscle, we set the Athletic Type variable to Elite—as recommended by the manufacturer for subjects who underwent regular training and present no visible folds of fat.

The thickness of subcutaneous adipose tissue was measured at 10 anatomical sites as follows: biceps, triceps, chest, scapula, axilla, waist, hip, thigh, medial calf, and lower back using the BodyMetrix device, as instructed by the manufacturer (Table 2). While performing the measurements, we relied entirely on the BodyViewProFit software’s automatic algorithm for identifying the fat–muscle interface. A pea-sized amount of ultrasound gel was applied by the examiner on the transducer head. Then, the transducer was placed in close contact with the skin, the measure button was pressed, and the examiner glided the transducer roughly 5 mm above and below the selected site throughout the measurement (5–8 s) while applying a minimal inward force to the transducer to avoid deforming the underlying tissue. Four formulas implemented in the BodyViewProFit were used to calculate the percentage of body fat: 3-sites Jackson and Pollock (JP3), 7-sites Jackson and Pollock (JP7), 9-sites Parrillo (P9), and 1-point biceps (B1). These are proprietary formulas. Although some of them are rooted in anthropometry (see [20] for JP3 and JP7, and [17] for P9), they were adapted by the research and development team of IntelaMetrix to express %BF in terms of the thicknesses of uncompressed adipose tissue instead of the thicknesses of skinfolds under standard stress exerted by the caliper’s jaws [7].

Each set of measurements started with the sites included in the JP7 formula and continued with the extra sites included in the P9 and B1 formulas, respectively. To compute %BF using JP3, P9, and B1, the adipose layer thicknesses were entered manually into the BodyViewProFit software.

### 2.3. Statistical Analyses

The experimental results are reported in this paper as the mean ± standard deviation (SD) and are shown in the figures as box plots or violin plots, as indicated. In the statistical hypothesis testing, the level of statistical significance was set to *p* < 0.05.

To pinpoint statistically significant differences between the mean %BF values obtained using different predictive formulas, we relied on the one-way analysis of variance (ANOVA) for the normally distributed data, and on the Kruskal–Wallis test for the data whose distribution deviated from normality according to the Shapiro–Wilk test. When statistical significance was reached in the ANOVA test, we performed pairwise comparisons using Scheffé’s method; when the Kruskal–Wallis test indicated significant differences between data sets, we applied Conover’s post hoc test [21].

We performed Passing–Bablok (PB) regressions to compare body composition assessments provided by different formulas. As opposed to least-squares linear regression, which assumes that the values of the predictor variable ($x_{i}$) are known beforehand, the PB regression was specifically designed to compare two data sets, $x_{i}$ and $y_{i}$, given by two different methods of measurement, both affected by experimental errors (here i=1, 2, …, n with n denoting the sample size) [22,23]. The slope, b, of the PB regression line (y=a+bx), is the median of the slopes of all the n(n−1)/2 lines that connect pairs of data points, $\left(x_{i},\;y_{i}\right)$—$\left(x_{j},\;y_{j}\right)$, with i<j. The intercept, a, is given by $a=\tilde{y}-b\tilde{x}$, where $\tilde{x}$ and $\tilde{y}$ denote the medians of $x_{i}$ and $y_{i}$, respectively. If zero is not part of the 95% confidence interval (CI) of a, one concludes that systematic differences exist between the data acquired by the two methods. If 1 does not belong to the 95% CI of b, there are proportional differences between the compared data sets. For these conclusions to be valid, Passing and Bablok recommended a sample size of at least 50 [23]. The PB method can only be applied if the data are well-fitted by a linear model; therefore, the CUSUM test for linearity was also performed.

Finally, we conducted a Bland–Altman analysis of body composition estimates given by different prediction formulas [24,25]. The differences between %BF given by two distinct formulas were plotted versus their mean (resulting in one data point per subject); then, a solid horizontal line was added to represent the mean of the differences (Mean), and dashed horizontal lines were added to plot the 95% interval of agreement, delimited by the lower limit of agreement, LLA = Mean−1.96 SD, and the upper limit of agreement, ULA = Mean + 1.96 SD (here, SD denotes the standard deviation of the differences). Note that Mean, LLA, and ULA are derived from our particular sample. Their 95% CIs, represented by error bars, show how precise these estimates are for the entire population of soccer players [24]. To point out proportional bias, encountered when the values given by one method diverge progressively from those of the other, we also performed a least squares linear regression analysis of differences versus means [26].

Unlike the Bland–Altman analysis, the PB regression is not sensitive to outliers. Therefore, we chose the PB regression over other sorts of regression procedures specifically designed for method comparisons, such as the major axis (or Deming) regression, or the ordinary least products regression [27]. Using both the Bland–Altman and PB methods, we sought to make sure that our conclusions are robust against outliers.

The statistical analyses and graphical representations were carried out using MedCalc version 20.015 (MedCalc Software Ltd., Ostend, Belgium).

## 3. Results

This section presents a comparative assessment of four prediction formulas, JP7, JP3, P9, and B1, for computing %BF from subcutaneous fat thicknesses measured in a group of male soccer players via AUS.

### 3.1. Descriptive Statistics and One-Way Analysis of Variance

Table 3 presents the descriptive statistics of the four data sets. The mean %BF provided by JP7 and JP3 are in remarkable agreement and differ from the ones given by P9 and B1. Whether these differences are statistically significant will be addressed later in this subsection.

**Figure 1:**
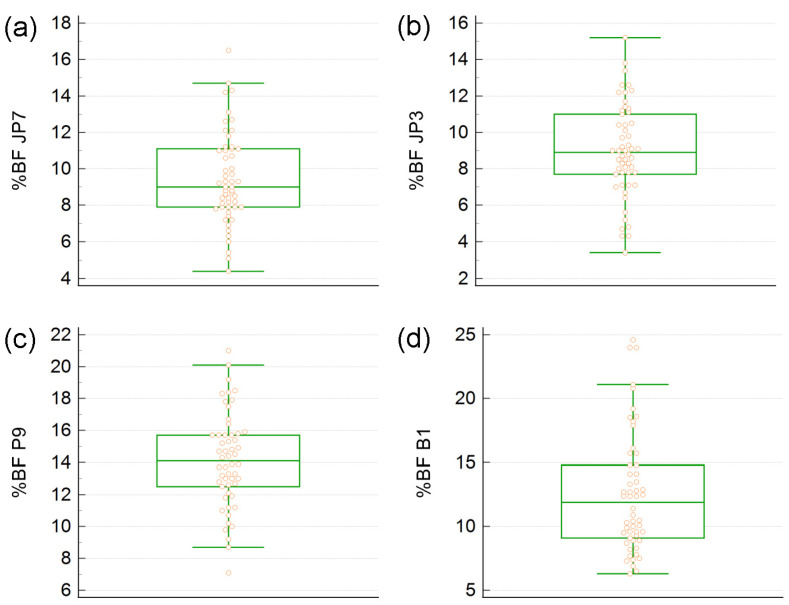
Box plots of body fat percentage (%BF) assessed by A-mode ultrasound using four different prediction formulas implemented in the BodyViewProFit software shipped with the BodyMetrix instrument: (**a**) the 7-sites Jackson–Pollock (JP7) formula, (**b**) the 3-sites Jackson–Pollock (JP3) formula, (**c**) the 9-sites Parrillo (P9) formula, and (**d**) the 1-point biceps (B1) formula. In each plot, the lower edge of the box depicts the first quartile (Q_1_); the horizontal line within the box represents the median, or the second quartile (Q_2_); and the upper edge of the box depicts the third quartile (Q_3_). The points beyond the whiskers are considered outliers.

Shown in Figure 1 are the box plots of %BF values given by the JP7 (a), JP3 (b), P9 (c), and B1 (d) formulas. The medians, represented by the horizontal lines that split the boxes, are similar for JP7 and JP3. Interestingly, JP3 gave the smallest number of outliers (Figure 1b), although it was found less reliable than JP7 in a previous study conducted on a sample of the general population [12]. The least reliable formula, B1, gave the largest number of outliers and the widest interquartile interval—the difference between the third and the first quartiles (Figure 1d).

**Figure 2:**
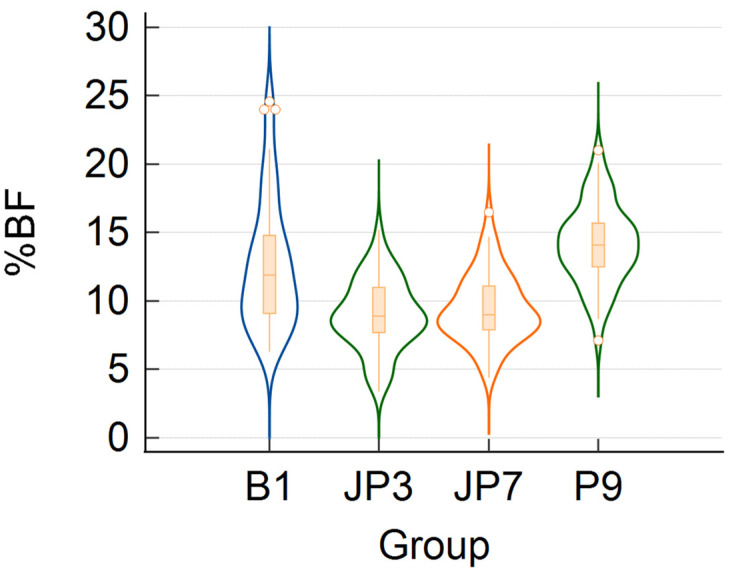
Violin plots of %BF values of the investigated group of 54 soccer players, obtained using A-mode ultrasound measurements of the subcutaneous fat thickness at certain anatomical sites and the B1, JP3, JP7, and P9 formulas (acronyms are explained in the caption of Figure 1). The width of the violin plot at a given %BF value tells how frequently it is encountered in the data set (wider regions correspond to more frequently occurring values).

The probability density functions of the four data sets are represented in the violin plots of Figure 2. The distributions of the %BF values given by the JP3 and JP7 formulas are strikingly similar and different from those derived from the other two formulas. Although Figure 2 shows deviations from the bell-shaped plot of the probability density function of the normal distribution, the *p*-values of the Shapiro–Wilk test indicate that the %BF obtained from JP3, JP7, and P9 are normally distributed, whereas those provided by B1 are not (Table 3, last column). Therefore, we conducted a one-way ANOVA test to find out whether there are statistically significant differences between the mean %BF given by JP3, JP7, and P9 (listed in Table 1). The test returned a *p*-value of less than 10^−3^, indicating that there are statistically significant differences between the means of at least two data sets. Pairwise comparisons based on Scheffé’s method revealed that the mean %BF given by the P9 formula differs from the other two means, which do not differ significantly from each other.

To compare all four data sets, we applied the Kruskal–Wallis test because, unlike ANOVA, it does not rely on the assumption of normally distributed data. It is a nonparametric test based on ranking the observed values and evaluates the validity of the null hypothesis that the compared data sets come from identical population distributions. According to the *p*-value of the Kruskal–Wallis test, *p* < 10^−6^, the null hypothesis should be rejected. Conover’s post hoc test of pairwise comparisons [21] points out that the JP3 and JP7 data sets are not significantly different, whereas the B1 and P9 data sets differ from all the others.

The conclusions drawn from the analysis of variance are reinforced by Lin’s concordance correlation coefficient (CCC), a dimensionless number ranging from 0 to 1 (the higher the better) [28]. CCC is a measure of both precision and accuracy. Between B1 and JP7, the CCC was 0.464, and the corresponding 95% CI was [0.328, 0.581]; between P9 and JP7, the CCC was 0.341 with 95% CI [0.238, 0.437]; finally, between JP3 and JP7, the CCC was 0.909 with 95% CI [0.850, 0.946].

### 3.2. Passing–Bablok Regression

**Figure 3:**
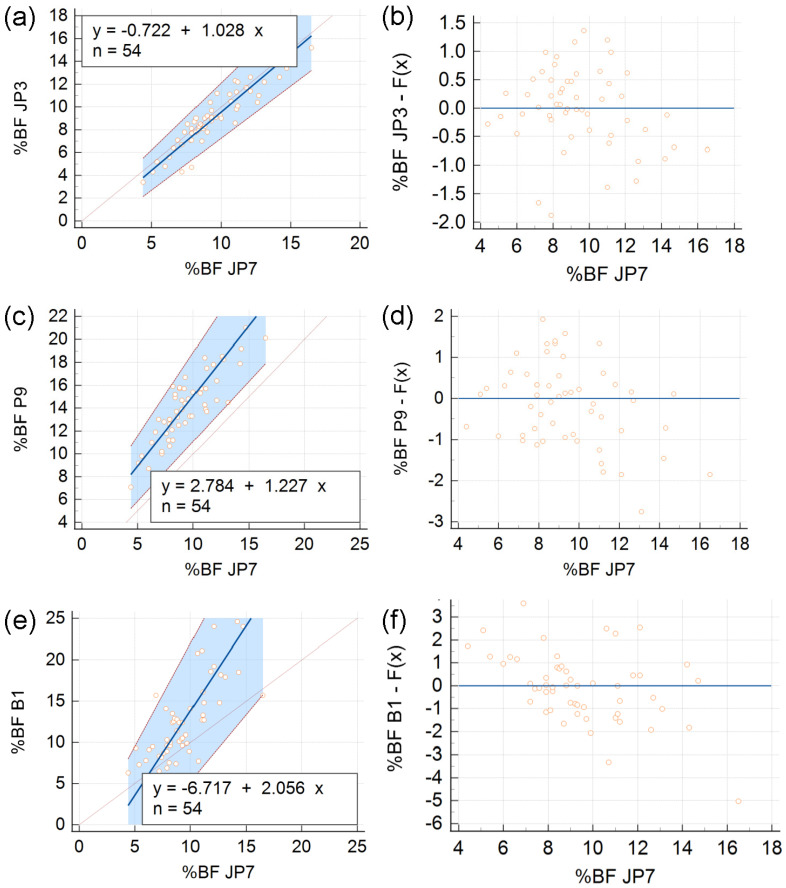
Method comparisons based on Passing–Bablok (PB) regression. Three prediction formulas (JP3, P9, and B1) are compared to the reference (JP7) formula; panels (**a**,**c**,**e**) show regression lines (thick solid lines) and give their equations, whereas (**b**,**d**,**f**) are plots of the corresponding residuals—differences between individual data points and the values predicted by the regression equation, *y* = *F*(*x*). In each of panels (**a**,**c**,**e**), the regression line is flanked by dotted lines depicting its confidence interval (shaded in blue); next to them, the identity line, *y* = *x*, is represented as a thin solid line.

Figure 3 represents the outcomes of PB regressions. Here, the JP7 data set is compared with JP3 (panels **a** and **b**), P9 (panels **c** and **d**), and B1 (panels **e** and **f**).

The *p*-values returned by the CUSUM test for linearity (0.72 for JP3 vs. JP7, 0.92 for P9 vs. JP7, and 0.22 for B1 vs. JP7) show that the PB regression is applicable.

When JP3 is compared to JP7 (Figure 3a), the intercept is -0.722 with 95% CI [−1.882, 0.316]. Since this interval contains 0, we conclude that there are no systematic differences between the two methods. The slope is 1.028 with 95% CI [0.914, 1.179], which means that the slope does not differ significantly from 1, so there are no proportional differences, either. The corresponding residuals are plotted in Figure 3b. The residual standard deviation (RSD) for this regression is 0.705. Thus, the interval that includes 95% of the residuals, given by [−1.96 RSD, 1.96 RSD] = [−1.382, 1.382], is relatively narrow.

When P9 is compared to JP7 (Figure 3c), the intercept is 2.784 and its 95% CI [0.592, 4.254] does not contain 0; the slope is 1.227 and its 95% CI [1.049, 1.462] does not include 1. Hence, the Passing–Bablok regression reveals both systematic and proportional differences between the %BF values given by P9 and JP7. For this regression, the RSD is 1.018, so 95% of the residuals lie between [−1.995, 1.995] (Figure 3d).

Finally, in the regression of B1 vs. JP7, the intercept is −6.717 with 95% CI [−10.90, −3.007] and the slope is 2.056 with 95% CI [1.618, 2.500]; the RSD is 1.536 and, therefore, 95% of the residuals belong to the interval [−3.01, 3.01], the widest of all three (Figure 3f).

### 3.3. Bland-Altman Analysis

**Figure 4:**
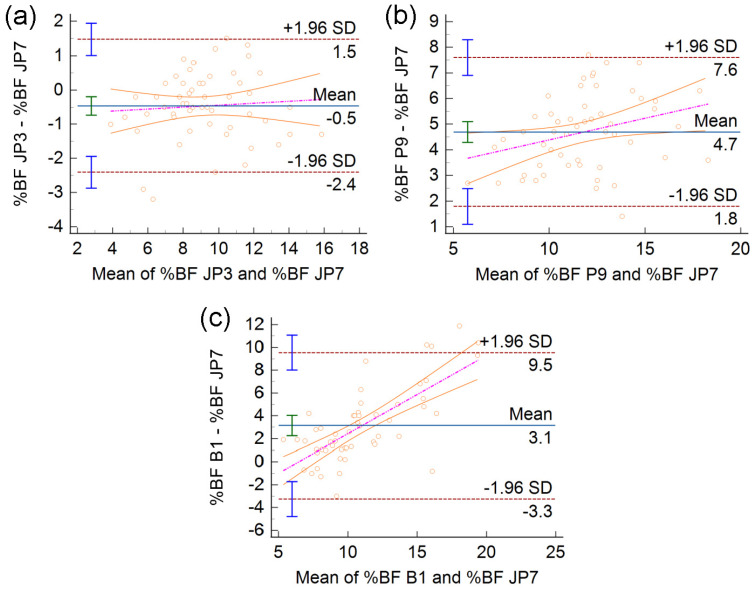
Bland–Altman plots of differences vs. means of %BF estimates provided by different prediction formulas. (**a**) JP3, (**b**) P9, and (**c**) B1 are compared to the reference formula, JP7. In each plot, the solid horizontal line depicts the mean of the differences, whereas dashed horizontal lines represent the upper limit of agreement (ULA = Mean + 1.96 SD) and the lower limit of agreement (LLA = Mean−1.96 SD); here, SD stands for the standard deviation of the differences. The error bar displayed on each horizontal line represents the 95% CI of the corresponding quantity. The dashed–dotted linear regression line, sandwiched between its 95% CI curves, is indicative of proportional bias whenever its slope is significantly different from zero.

In the Bland-Altman (BA) plots from Figure 4, each marker results from two tests applied to the same subject, and its position with respect to the horizontal axis is a measure of the subject’s global adiposity. The linear regression analysis of differences vs. means reveals the presence of proportional bias in Figure 4, panels **b** and **c**, dashed–dotted lines.

According to Figure 4a, JP3 slightly underestimates the global adiposity of soccer players in comparison to JP7. In contrast, P9 provides a significant overestimation of %BF, especially in subjects with higher than average %BF (Figure 4b). The B1 formula agrees well with JP7 in the case of extremely lean athletes and deviates progressively from JP7 for subjects of higher adiposity (Figure 4c). The width of the 95% interval of agreement (ULA–LLA) is the smallest for JP3 compared to JP7 (3.9 %BF), intermediate for P9 compared to JP7 (5.8 %BF), and the largest for B1 compared to JP7 (12.8 %BF).

## 4. Discussion

This paper presents a comparative study of four prediction formulas for A-mode ultrasound (AUS) assessment of body fat percentage (%BF) in male soccer players. The reported results confirm our working hypothesis, that JP3 and JP7 agree well and deviate significantly from P9 and B1.

Conducted on a sample of professional soccer players, this work complements previous investigations of male athletes using the BodyMetrix AUS instrument [11]. Wagner et al. established that, relying on AUS measurements of subcutaneous adipose tissue thicknesses at the chest, abdomen, and thigh, the JP3 formula gives an accurate estimate of %BF in Division I male collegiate athletes in comparison to the BOD POD body composition tracking system. More precisely, JP3 overestimated the mean %BF of men by about 1.5 %BF, which was statistically insignificant [11]. Based on several methods of statistical analysis, the present study indicates that JP3 and JP7 are in excellent agreement. Compared to JP3, JP7 results in an additional overestimation of the adiposity of athletes, on average by 0.5 %BF (Figure 4a). Nevertheless, zero lies close to the upper limit of the 95% CI of the bias (Mean), suggesting that this mean difference is just marginally significant. Moreover, the observed bias is smaller than the technical error of measurement of the BodyMetrix instrument [11,12]. Thus, it seems safe to conclude that the validity of JP3 also implies the validity of JP7 in the athletic population.

The question arises, why should we take seven AUS measurements when three will do? In a previous investigation of the reliability of AUS [12], we found that JP7 assured better precision than JP3 when the BodyMetrix device was used in full-automatic mode—i.e., when the operator never overrode the BodyView software in spotting the maximum that corresponds to the fat–muscle interface. The 3-sites Pollock and JP3 had roughly the same reliability, whereas the B1 formula was the least reliable, suggesting that precision is favored by taking measurements at more anatomic sites [12].

The P9 formula was a strong candidate in our study, not just from the reliability perspective, but also because of its origin—it was specifically designed for %BF assessments of bodybuilders based on skinfold thickness data [17]. To our knowledge, this is the first work to evaluate the P9 formula for testing the body composition of athletes via AUS. Our results, obtained for professional soccer players, agree surprisingly well with those reported in the literature for a heterogeneous sample of men [16]. Compared to JP7, we found that P9 overestimates the adiposity of male athletes by 4.7 %BF, on average (Figure 4b). In the work by Lowry et al., the mean difference between the BOD POD and AUS was −0.94 %BF for JP3, −1.23 %BF for JP7, and −4.72 %BF for P9 [16]. Note that, just like in athletes, in males from the general population, both JP3 and JP7 result in a slight overestimation of fatness compared to ADP, whereas P9 gives significantly higher %BF values [16].

The B1 formula was evaluated in this study because it is convenient for both the subject and the examiner. Although it was originally devised to assess the body fat percentage of morbidly obese people in a non-embarrassing manner, the B1 formula gave reasonable estimates only in the case of extremely lean members of our study group, with 5 to 10 %BF (Figure 4c). Using B1, the test–retest reliability of AUS was deemed better than that of JP3 in a study conducted on 11 relatively lean college students (18.2 ± 8.3 %BF, according to JP3) [18], and relatively poorer in another one, performed on a sample of 144 adults with BMI = 24.6 ± 4.7 kg/m^2^, ranging from 16.6 to 45 kg/m^2^ [12]. The source of this discrepancy is unclear, but differences in the AUS measurement procedure or sample characteristics might have contributed to it. If a highly reliable measurement protocol will emerge for AUS using B1, in agreement with [18], the present study indicates that the B1 formula could be used to rapidly assess the adiposity of lean athletes.

The limitations of this work include the lack of explicit validation of the investigated prediction formulas and the exclusive focus on soccer players. Further research is warranted to firmly establish the validity of AUS prediction formulas in a diverse athletic population. Future studies could test the accuracy of AUS against the four-component (4C) model, based on ADP or underwater weighing for measuring body volume, deuterium dilution or bioelectrical impedance spectroscopy for evaluating the amount of total body water, and DXA for assessing bone mineral content [29,30].

The characterization of nutritional status via AUS deserves further scrutiny in light of recent advances in brightness (B)-mode ultrasound for testing the adiposity of athletes, devised under the supervision of the Medical Commission of the International Olympic Committee [31,32,33]. Ultrasound methods have emerged as alternatives to skinfold thickness measurements and, therefore, focused on the same anatomic sites. A thorough knowledge of anatomy and considerable practice are needed to locate them [7]. Furthermore, some of them do not allow for clear ultrasound images. The waist site, for instance, is midway between the umbilicus and the abdominal skinfold site [7], which was found challenging in a previous investigation by B-mode ultrasound: the presence of Camper’s fascia made it difficult to identify the borders of the subcutaneous adipose tissue layer [34]. Therefore, eight standard sites (three on the trunk, two on the right arm, and three on the right leg) were proposed for subcutaneous adipose tissue patterning via diagnostic ultrasound. Defined in terms of the subject’s height, they can be marked accurately with minimal (1 h) training, and the underlying adipose tissue thickness is roughly uniform around them [32]. The new methodology turned out to be highly reliable over a wide range of body compositions in single- [33] and multi-centric [31] studies. Moreover, prediction formulas are being developed for assessing body composition based on ultrasound data collected at these standard sites, with the 4C model as a criterion measure [31].

In conclusion, this article presented a comparative evaluation of four prediction formulas implemented in the BodyViewProFit software bundled with the BodyMetrix A-mode ultrasound device. The reported findings suggest that JP3 and JP7 are equally accurate in estimating the body fat percentage of male athletes, whereas P9 and B1 overestimate their adiposity. JP3 is more convenient when subject comfort and test duration are matters of concern; JP7 is recommended when precision is the most important aspect and the ultrasound instrument is used in the automatic mode.

## Figures and Tables

**Table 1:** Subject characteristics (*n* = 54).

	Mean ± SD ^1^	Min	Max	Q_1_	Q_2_	Q_3_
Age (y)	22.9 ± 3.83	18	32	20	22	25
Height (m)	1.82 ± 6.01	1.73	1.95	1.77	1.82	1.86
BM (kg)	75.5 ± 6.04	65	90	71	74.5	80
BMI (kg/m^2^)	22.8 ± 1.39	19.5	26.1	21.7	22.7	23.8

^1^ Abbreviations: SD—standard deviation; BM—body mass; BMI—body mass index = BM (kg) divided by height (m) squared; Q_1_—first quartile (25% of the values lie below it); Q_2_—second quartile, or median (half of the values lie below it); Q_3_—third quartile (75% of the values lie below it).

**Table 2:** Anatomic sites for subcutaneous adipose tissue layer thickness measurements required by the evaluated formulas, as instructed by the BodyViewProFit software.

Site	Description	Formulas ^1^
Biceps	midway between the tip of the shoulder and the elbow along the anterior midline	B1, P9
Triceps	midway between the tip of the shoulder and the elbow along the posterior midline	JP7, P9
Chest	midway between the axillary line of the underarm and the nipple	JP7, JP3, P9
Scapula	just below the bottom tip of the shoulder blade at the inferior angle	JP7, P9
Axilla	on the midaxillary line and level with the lower tip of the breastbone (Xiphoid Process)	JP7
Waist	one inch (2.54 cm) to the right of the umbilicus	JP7, JP3, P9
Hip	just above the iliac crest (front tip of the top of the hip bone)	JP7, P9
Thigh	midway between the proximal border of the kneecap and the crease of the hip	JP7, JP3, P9
Lower Back	over the kidney and two inches to the right of the spine	P9
Calf	at the maximum circumference of the calf, midline of its medial inside border	P9

^1^ Abbreviations: B1—1-point biceps, JP3—3-sites Jackson and Pollock [20], JP7—7-sites Jackson and Pollock [20], P9—9-sites Parrillo [17].

**Table 3:** Descriptive statistics of %BF data (*n* = 54) obtained from the JP7, JP3, P9, and B1 formulas.

Formula	Mean ± SD	Median	Min	Max	Skewness	Kurtosis	*p* ^1^
JP7	9.45 ± 2.51	9.0	4.4	16.5	0.556	0.321	0.290
JP3	8.99 ± 2.58	8.9	3.4	15.2	0.031	−0.167	0.846
P9	14.1 ± 2.94	14.1	7.1	21.0	0.050	−0.065	0.994
B1	12.6 ± 4.72	11.9	6.3	24.6	0.950	0.237	8 × 10^−4^

^1^ *p*-value associated with the Shapiro–Wilk test for normal distribution.

## Data Availability

**The** data collected in the course of this investigation have been anonymized and shared as supporting information (see Supplementary Materials).

## Supplementary Materials

The following supporting information is available online at: https://www.mdpi.com/article/10.3390/diagnostics13040690/s1, Data S1: Anonymized raw data file.
